# Topical application of *Cinnamomum* hydroethanolic extract improves wound healing by enhancing re-epithelialization and keratin biosynthesis in streptozotocin-induced diabetic mice

**DOI:** 10.1080/13880209.2019.1687525

**Published:** 2019-11-23

**Authors:** Amin Daemi, Mahsa Lotfi, Mohammad Reza Farahpour, Ahmad Oryan, Sina Jangkhahe Ghayour, Ali Sonboli

**Affiliations:** a Department of Medical Biochemistry, Faculty of Medicine, Cukurova University, Adana, Turkey;; b Faculty of Pharmacy, Tabriz university of Medical Sciences, Tabriz, Iran;; c Department of Clinical Sciences, Faculty of Veterinary Medicine, Urmia Branch, Islamic Azad University, Urmia, Iran;; d Department of Pathology, School of Veterinary Medicine, Shiraz University, Shiraz, Iran;; e Department of Biology, Medicinal Plants and Drugs Research Institute, Shahid Beheshti University, G.C. Evin, Tehran

**Keywords:** Antioxidant activity, cinnamaldehyde, insulin-like growth factor 1, glucose transporter-1

## Abstract

**Context:** *Cinnamomum verum* J. Presl. (Lauraceae) has a high number of polyphenols with insulin-like activity, increases glucose utilization in animal muscle, and might be beneficial for diabetic patients.

**Objective:** This study evaluated the effectiveness of an ointment prepared from *Cinnamomum verum* hydroethanolic extract on wound healing in diabetic mice.

**Materials and methods:** A total of 54 male BALB/c mice were divided into three groups: (1) diabetic non-treated group mice that were treated with soft yellow paraffin, (2 and 3) mice that were treated with 5 and 10% *C. verum*. Two circular full-thickness excisional wounds were created in each mouse, and the trial lasted for 16 d following induction of the wound. Further evaluation was made on the wound contraction ratio, histopathology parameters and mRNA levels of cyclin D1, insulin-like growth factor 1 (IGF-1), glucose transporter-1 (GLUT-1), total antioxidant capacity, and malondialdehyde of granulation tissue contents. HPLC apparatus was utilized to identify the compounds.

**Results:** The HPLC data for cinnamon hydroethanolic extract identified cinnamaldehyde (11.26%) and 2-hydroxyl cinnamaldehyde (6.7%) as the major components. A significant increase was observed in wound contraction ratio, fibroblast proliferation, collagen deposition, re-epithelialization and keratin biosynthesis in the *C. verum*-treated groups in comparison to the diabetic non-treated group (*p* < 0.05). The expression level of cyclin D1, IGF1, GLUT 1 and antioxidant capacity increased in the *C. verum*-treated groups in comparison to the diabetic non-treated group (*p* < 0.05).

**Conclusions:** Topical administration of *C. verum* accelerated wound healing and can possibly be employed in treating the wounds of diabetic patients.

## Introduction

Under normal circumstances, wound healing has different stages including proliferation and migration to the wound site, appropriate angiogenesis, re-epithelialization, and proper biosynthesis (Daemi et al. 2019; Manzuoerh et al. 2019). Diabetes inhibits cellular infiltration, angiogenesis and attenuation of wound contraction and closure (Bonab and Farahpour 2017). During diabetes, hyperglycaemia delays wound healing due to the increase in oxidative stress and decrease in insulin-like growth factor 1 (IGF-1) expression. IGF-1 has a stimulator effect on keratinocyte and fibroblast proliferation and re-epithelialization, improving the effects on wound strength (Blakytny and Jude 2006; Daemi et al. 2019). The decrease in endothelial insulin/IGF-1 signalling is a key factor that faults wound healing in mice (Aghdam et al. 2012).

Glucose transporters (GLUTs) are also involved in transporting the glucose into various cell types. Insulin not only regulates glucose uptake by GLUT-1 (Bonab and Farahpour 2017; Oryan and Alemzadeh 2017; Daemi et al. 2019), but it also increases the total levels and expression of GLUT1 in the plasma membrane of the basal cells in skin. Cyclin D1 regulates the cell cycle transition by G1, and its over-expression promotes the cell cycle via progressing the G1 phase of the cell cycle during cell proliferation, further stimulating contact-independent cellularity (Tashiro et al. 2007).

Obtained from the inner bark of trees, *Cinnamomum verum* J. Presl. (Lauraceae) (cinnamon) has medicinal and culinary uses (Farahpour and Habibi 2012; Mohammadi et al. 2014). It contains considerable levels of polyphenols with insulin-like activity and enhances glucose utilization in animal muscle tissue which reduces sugar (Mang et al. 2006). This study evaluated the effectiveness of a new ointment formulation, prepared from the cinnamon hydroethanolic extract (*C. verum* ointment), on wound healing in diabetic mice.

## Materials and methods

### Chemicals

All the HPLC grade solvents including phosphoric acid, acetonitrile HPLC-grade, methanol, ultra-pure water and also malondialdehyde and butylated hydroxytoluene were purchased from Merck (Darmstadt, Germany). In addition, streptozotocin was purchased from the Sigma-Aldrich Company (St. Louis, MO).

### Preparation of the plant extract

Powdered *C. verum* bark was purchased (in December 2016) from a local market in Mumbai City, India, and authenticated by Dr Sonboli, a botanist. A voucher specimen (MPH-2880) has been deposited in the Medicinal Plant and Drugs Research Institute Herbarium (MPH), Shahid Beheshti University, Tehran, Iran. The bark was extracted as described by Farahpour et al. (2017).

### HPLC analysis

HPLC analyses were conducted using a KNAUER system (PLATINblue) equipped with a photodiode array (PDA) detector and an auto-sampler. EZChrom Elite software was used for analyzing the data. All the separations were conducted on ODS-2 C18 (250 × 4.6 mm) column and experiments were done at room temperature. The column was eluted by a mobile phase composition of solvent A, 0.1% phosphoric acid and solvent B, acetonitrile as follows: 0 min, 10% A; 12 min, 87% A; 8 min, 67% A; 10 min; after maintaining the solvent at this composition for 2 min, eluent A was reduced by 57% over the next 10 min, and then held at this level until the end of the 60 min analysis. The flow rate of the mobile phase was 1.0 mL/min. The chromatogram was monitored at a wavelength of 265 nm throughout the experiment.

### Assessment of 2,2-di (4-tert-octylphenyl)-1-picrylhydrazyl (DPPH)

Free radicals scavenging activity assay (FRSA) was determined as previously described by Farahpour et al. (2015). Briefly, the DPPH radicals scavenging activity was calculated as follows:

$$\text{FRSA}=\left[\left(A_{0}-A_{1}/A_{0}\right)\times 100\right]$$

where A_0_ is the absorbance of the control (blank, without extract) and A_1_ is absorbance in the presence of extract or standard sample.

### Assessment of total phenolic content

Total phenolic contents (TPC) of the cinnamon extract were characterized using the Folin–Ciocalteu method as already published by Farahpour et al. (2015). TPC in cinnamon extract was calculated as gallic acid equivalents (GAE) from a calibration curve (5, 20, 40, 60, 80, and 100 mg/mL) and presented as mg GAE/100 g dry weight of extract (mg GAE/100 g DW).

### Experimental design and induction of diabetes

Diabetes was induced by intraperitoneal administration of streptozotocin (60 mg/kg body weight in 0.01 M citrate buffer, pH 4.5) for five consecutive days in 54 healthy male BALB/c mice (12 weeks old). This study was approved by the Animal Research Committee of the Urmia Islamic Azad University with Ethical no. IAUUB 1122. The serum level of glucose was analyzed 5 d following the last administration of streptozotocin, and was then closely monitored for 2 weeks. The animals with fasting blood glucose levels higher than 300 mg/dL were considered as diabetic, and the wounds were then induced.

### Wound healing activity

All the diabetic mice (*n* = 54) were anaesthetized by intraperitoneal administration of ketamine and xylazine hydrochloride. Since the histological and molecular samples were taken on the same day, two 5 mm diameter circular full thickness wounds were created with a biopsy punch on the dorsal surfaces of each mouse (Farahpour and Habibi 2012). After induction of wound, all the animals were divided into three groups of 18 animals each, including diabetic non-treated group (or topically treated with soft yellow paraffin), and two treatment groups composed of two different concentrations of 5% and 10% *C. verum* mixed with white petrolatum. The ointments were topically applied once/day for 14 consecutive days. The wound area was measured on days 3, 6, 9 and 14 by a graph sheet (Bonab and Farahpour 2017). Wound closure percentage was evaluated using the original and final area drawn on glass slides during the experiments as presented below: Percentage of wound closure = [(wound area on day 0 − wound area on day X)/wound area on day 0] × 100.

### Histological and histomorphometrical studies

Following 3, 7 and 14 d after wound creation, full thickness cutaneous tissue samples were excised from the wound area. The specimens were then fixed in 10% neutral-buffered formalin, routinely processed, embedded with paraffin wax, sectioned at 5 µm thickness and stained with Masson’s trichrome for collagen intensity and immunofluorescent standing for keratin. The slides were examined by an ordinary light microscope (Olympus, CX31RBSF, Japan) so as to evaluate the predominant stage of wound healing. Three consecutive sections were obtained from each specimen. Inflammatory cell infiltration, fibroblasts and fibrocytes count, the number of blood vessels/mm^2^ of the tissue, oedema, collagen deposition, and tissue organization and alignment were also evaluated. The results for oedema scores and collagen intensity were reported in semi-quantitative format including negative (−), mild (+), mild to moderate (++), moderate (+++) and intensive (++++). The epithelial thickness was finally assessed by morphometric lens (Olympus, Germany) (Bonab and Farahpour 2017).

### Molecular analysis

DNA extraction and PCR conditions were run as follows: general denaturation at 95 °C for 3 min, 1 cycle, followed by 40 cycles of 95 °C for 20 s; annealing temperature (56 °C for IGF-1, 58 °C for cyclin D1, 58 °C for GLUT-1) for 30 s; elongation: 72 °C for 1 min and 72 °C for 5 min. The primers sequences were GAPDH, forward (5′-ACCACAGTCCATGCCATCAC-3′) and reverse (5′-CACCACCCTGTTGCTGTAGCC-3′); GLUT-1, forward (5′-GCCTGAGACCAGTTGAAAGCAC-3′) and reverse (5′-CTGCTTAGGTAAAGTTACAGGAG-3′); IGF-1, forward (5′-TAGGTGGTTGATGAATGGT-3′) and reverse (5′-GAAAGGGCAGGGCTAAT-3′); cyclin D1, forward (5′-ACCACAGTCCATGCCATCAC-3′) and reverse (5′-CACCACCCTGTTGCTGTAGCC-3′) (Farahpour et al. 2018).

### Antioxidant capacity of cinnamon

The wound granulation tissue was homogenized in ice-cold KCl (150 mM) and the mixture was then centrifuged at 3000 *g* for 10 min. The supernatant was used to evaluate the total antioxidant capacity (TAC), malondialdehyde (MDA) and total tissue thiol molecules (TTM) content. The MDA content and Lowry method of the collected granulation tissue were employed to determine the lipid peroxidation rate and protein content of the samples, respectively. In order to evaluate the TTM, the collected granulation tissue sample was homogenized in ice-cold KCl (150 mM), and the mixture was then centrifuged at 3000 *g* for 10 min. Thereafter, 0.5 mL of the supernatant was added to 0.6 mL Tris-EDTA buffer (Tris base 0.25 M, EDTA 20 mM, pH = 8.2) followed by addition of 40 μL DTNB (10 mM in pure methanol) in a 10 mL glass test tube.

### Statistical analyses

Two-way ANOVA was used for analyzing the results. Dunnett’s test for pair-wise comparisons was used for assessing the effect of time and treatments. Differences were considered significant if *p* < 0.05.

## Results

### Total phenolic contents and antioxidant activity

The antioxidant property of plant extracts is mainly due to their polyphenolic compounds. Total phenolic content of cinnamon extract was 300.53 mg/g dried extract. The results showed a considerable high number of DPPH radical scavenging activity related to cinnamon extract (23.61 µg/mL) compared to the butylated hydroxytoluene (20.32 µg/mL).

### HPLC analysis

**Figure 1.**
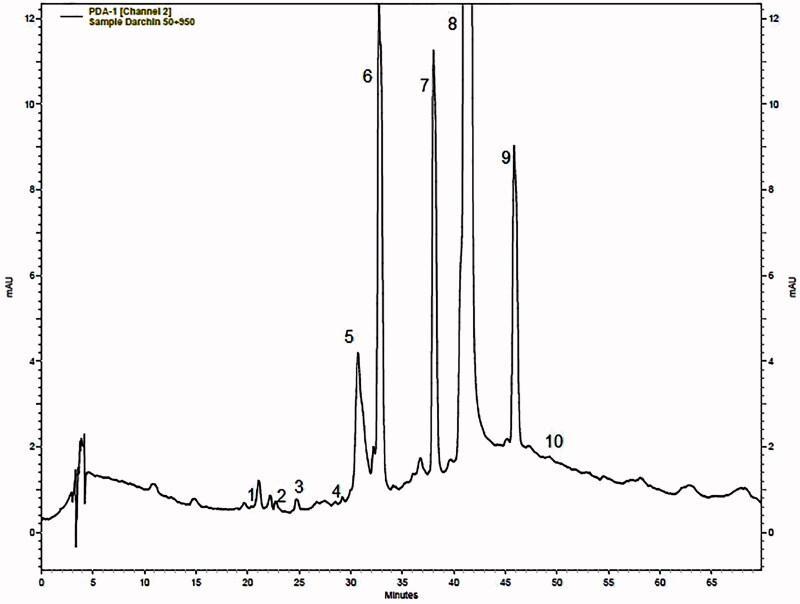
Representative chromatogram of a cinnamon extract on a ODS-2 C18 column. Peaks: (1) caffeic acid, (2) epicatechin, (3) coumarin, (4) quercetin (5) 2-hydroxyl cinnamaldehyde, (6) cinnamyl alcohol, (7) cinnamic acid, (8) cinnamaldehyde, (9) 2-methoxy cinnamaldehyde and (10) Eugenol.

The chromatogram obtained on an ODS-2 C18 column for the hydromethanolic extract of cinnamon is presented in Figure 1. From the HPLC chromatographic profile (Table 1), 10 compounds were determined on the basis of their retention time and through comparing the data with those of the pure standards. As shown in Table 1 analysis of the cinnamon extract resulted in the characterization of cinnamaldehyde (11.26%) and 2-hydroxy cinnamaldehyde (6.7%) as the principal components.

**Table 1.** Phenolic compounds content in cinnamon hydroethanolic extract.

Number	Compound	Amount (mg/g dry extract)
1	Caffeic acid	0.06
2	Epicatechin	0.01
3	Quercetin	0.02
4	Coumarin	0.10
5	2-Hydroxyl cinnamaldehyde	6.70
6	Cinnamyl alcohol	0.33
7	Cinnamic acid	0.27
8	Cinnamaldehyde	11.26
9	2-Methoxy cinnaldehyde	0.78
10	Eugenol	0.01

### Wound healing activity

The wound area was significantly reduced (*p* < 0.05) in both *C. verum*-treated groups (Table 2) compared with the diabetic non-treated group animals on days 6, 9 and 14 following wound creation.

**Table 2.** Effects of *C. verum* on circular excision wound area (mm^2^) in diabetic mice.

Groups	Wound area (mm^2^) ± S.D.				
	0	3	6	9	14
Control	19.63 ± 0.0	19.43 ± 2.17^a^	17.25 ± 3.20^a^	7.90 ± 1.41^a^	5.63 ± 0.32^a^
5% *C. verum*-treated	19.63 ± 0.0	14.93 ± 1.62^b^	12.99 ± 1.46^b^	6.21 ± 0.56^b^	1.09 ± 0.35^b^
10% *C. verum*-treated	19.63 ± 0.0	13.87 ± 1.76^b^	10.32 ± 0.94^c^	3.22 ± 0.16^c^	0.33 ± 0.08^b^

*n* = 6 animals in each group. Data are presented as the mean ± S.D. There are significant differences between groups with different superscripts (^a,b,c^*p* < 0.05 versus Controls).

### Histopathology study

**Figure 2.**
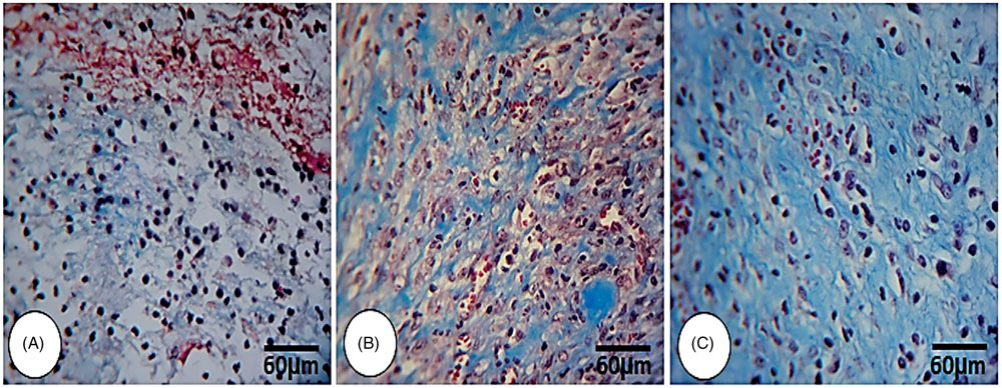
Histological photomicrograph from wound area on day 3. (A) Control group, (B) 5% *C. verum*-treated and (C) 10% *C. verum*-treated groups. Note well-formed granulation tissue, high collagen biosynthesis as well as condense collagen condensation in *C. verum*-treated groups, (Masson-Trichrome staining, 600×).

**Figure 3.**
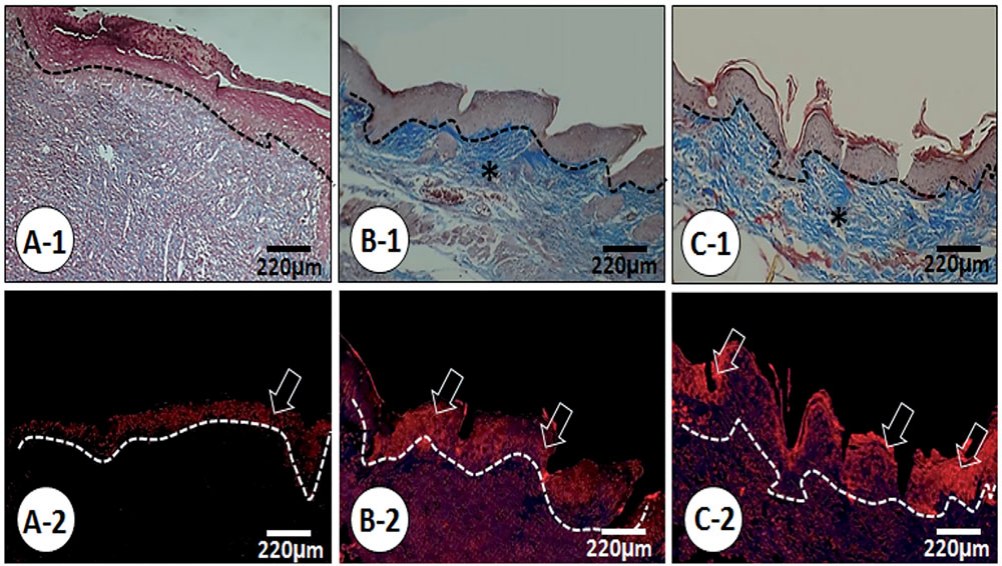
Histological photomicrograph from wound area on day 14. (A) Control group, (B) 5% *C. verum* treated and (C) 10% *C. verum*-treated groups. Note well-formed collagen condensation (*) in dermis of the *C. verum*-treated groups versus non-treated Control animals. The Masson-trichrome stained cross sections are representing complete re-epithelialization in *C. verum*-treated groups, on day 14. In the second row, the special fluorescent staining for keratin-6 are represented, which are indicating a significant enhancement in keratin biosynthesis in *C. verum*-treated groups, (400× magnification).

The animals in the *C. verum*-treated groups showed lower oedema score and fewer inflammatory cell infiltration compared to the diabetic non-treated group animals, seven and fourteen days after wound creation (*p* < 0.05) (Table 3). Animals in the 10% *C. verum*-treated group exhibited significantly (*p* < 0.05) higher new blood vessel formation (Table 3) and well-formed granulation tissue (Figure 2) versus 5% *C. verum*-treated animals, seven days after wound creation. The re-epithelialization, up to five layers, was initiated but the diabetic non-treated group showed thinner layers of epithelial cells, 1–3 layers, on day seven following wound creation. Complete re-epithelialization was observed 14 d after *C. verum* administration, yet weaker and fewer cellular layers were observed in the diabetic non-treated group. Following the evaluation of keratin biosynthesis by a special immunofluorescence staining for keratin K-6 (Figure 3), it was observed that the synthesis of keratin significantly increased in the 5% and 10% *C. verum*-treated animals in comparison to the diabetic non-treated group, 14 d after wound creation.

**Table 3.** Effects of *C. verum* on mean distribution of fibroblasts, fibrocytes per one mm^2^ of the wound tissue and epithelium thickness (µm) and oedema in different groups on different days.

Groups	Oedema	Immune cell	Vessels	Fibroblast	Collagen	Epithelium
Day 3						
Control	++++	105 ± 5.98^a^	0.22 ± 0.21^a^	0.5 ± 0.29^a^	–	–
5% *C. verum*-treated	+++	82.8 ± 4.99^b^	6.5 ± 1.29^b^	4.50 ± 1.29^b^	+	15.25 ± 4.11^a^
10% *C. verum*-treated	+++	78.25 ± 5.91^b^	7.75 ± 0.96^b^	6.50 ± 1.29^b^	+	24.95 ± 9.71^b^
Day 7						
Control	+++	84.1 ± 6.47^a^	1.8 ± 0.85^a^	6.10 ± 1.84^a^	+	11.95 ± 1.17^a^
5% *C. verum*-treated	++	60.2 ± 3.86^b^	14 ± 2.71^b^	18.25 ± 2.50^b^	++	69.25 ± 4.57^b^
10% *C. verum*-treated	+	45.9 ± 3.42^c^	20.25 ± 2.63^c^	23.75 ± 2.63^b^	+++	95.50 ± 3.10^c^
Day 14						
Control	++	67.2 ± 3.12^a^	4.5 ± 1.11^a^	5.00 ± 0.80^a^	+++	78.50 ± 4.35^a^
5% *C. verum*-treated	+	40.75 ± 3.6^b^	8±−.82^b^	12.75 ± 2.21^b^	++++	162.75 ± 34.13^b^
10% *C. verum*-treated	–	27.5 ± 2.22^c^	8.25 ± 1.26^b^	14.75 ± 3.40^b^	++++	208.50 ± 36.68^b^

*Note*: Data are presented as the mean ± S.D. There are significant differences between groups with different superscripts (^a,b,c^*p* < 0.05 versus controls).

### Molecular analysis

**Figure 4.**
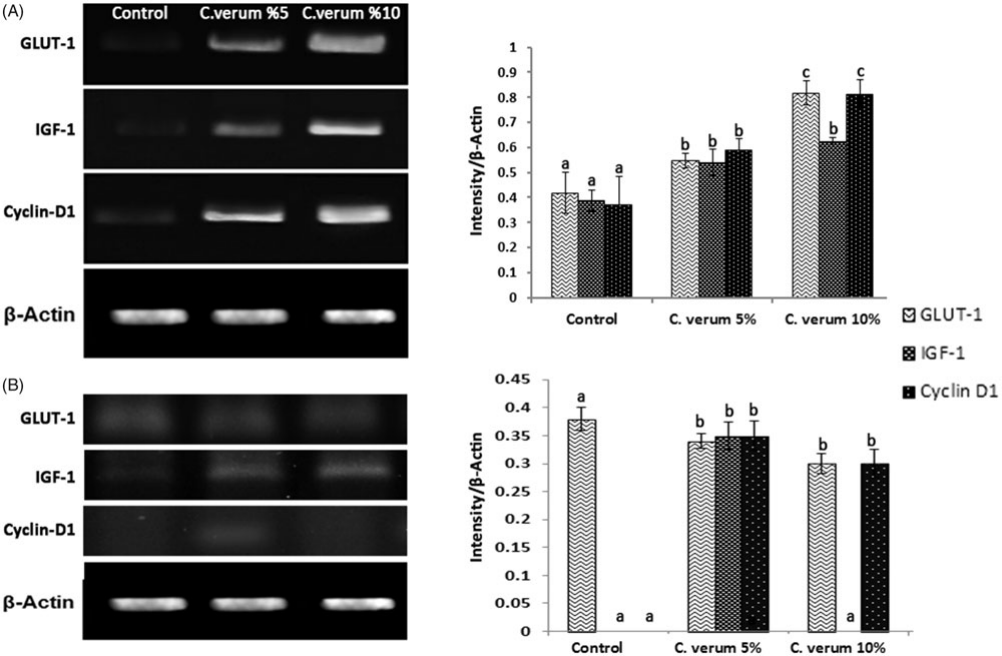
mRNA expression of IGF-1, cyclin D1 and GLUT-1 in different tests and control groups at 7 (A) and 14 (B) days after wound creation. Topical administration of *C. verum* elevated IGF-1, cyclin D1 and GLUT-1 mRNA expression in comparison with the control group, only at 7 d after wound creation (A). IGF-1, cyclin D1 and GLUT-1/β-actin intensity.

RT-PCR analyses showed that the administration of *C. verum* significantly (*p* < 0.05) enhanced IGF-1, GLUT-1 and cyclin D1 mRNA expression compared to the diabetic non-treated group on day 7 following wound creation (Figure 4(A)). More analyses did not show significant differences between the groups regarding GLUT-1; further observation was the diminished expression of cyclin D1 in the 10% *C. verum*-treated group (Figure 4(B)) on day 14.

### Biochemical estimations

Observations demonstrated that *C. verum* at higher doses, namely 5% and 10%, increased tissue TAC level on days 3 and 7 following wound creation in comparison to the diabetic non-treated group (Table 4). Analyses of tissue TTM revealed that 10% *C. verum* more significantly (*p* < 0.05) up-regulated the tissue TTM level (Table 3). Administration of the *C. verum* extract reduced (*p* < 0.05) the MDA level compared with the diabetic non-treated group on days 3 and 7 following wound creation (Table 4).

**Table 4.** Effects of *C. verum* on mean alteration of tissue TAC, MDA and TTM in different groups.

Groups	TAC (mmol Trolox Equiv. /L)	MDA (nmol/mg protein)	TTM (nM/mg protein)
Day 3			
Control	0.24 ± 0.05^a^	1.15 ± 0.57^a^	0.51 ± 0.04^a^
5% *C. verum*-treated	0.52 ± 0.04^b^	0.55 ± 0.07^b^	0.88 ± 0.04^b^
10% *C. verum*-treated	0.58 ± 0.04^b^	0.45 ± 0.04^c^	1.06 ± 0.15^c^
Day 7			
Control	0.33 ± 0.05^a^	0.68 ± 0.11^a^	0.64 ± 0.07^a^
5% *C. verum*-treated	0.58 ± 0.04^b^	0.36 ± 0.04^b^	1.18 ± 0.20^b^
10% *C. verum*-treated	0.72 ± 0.03^c^	0.26 ± 0.03^c^	1.32 ± 0.02^c^

*Note*: All data are presented in mean ± SD.

^a,b,c^ are representing significant differences between marked data in the same column (^a,b,c^*p* < 0.05 versus control-sham).

## Discussion

**Figure 5.**
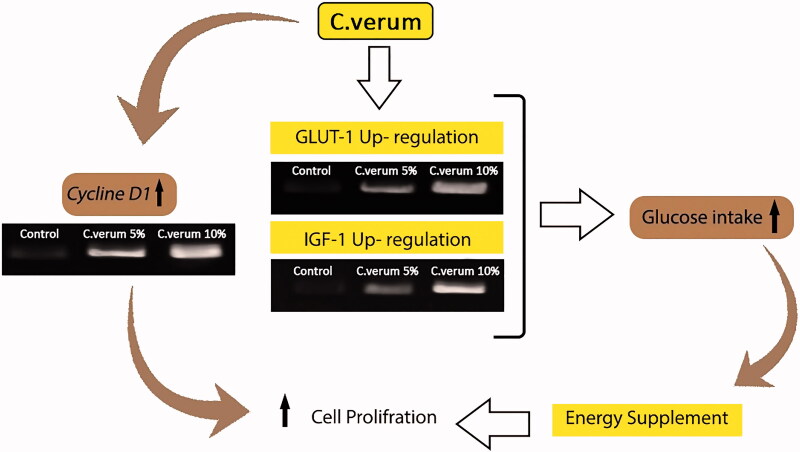
Promoting effect of topical administration of *C. verum* on wound healing process in diabetic animals. *C. verum* up-regulates the IGF-1 and GLUT-1 expression which in turn results in accelerated glucose transition into cells, and follow it faster granulation formation, re-epithelization and keratin biosynthesis.

Topical treatment with *C. verum* significantly inhibited lipid peroxidation ratio due to the increase in tissue antioxidant status by up-regulating TAC. The decrease in the tissue MDA level in the *C. verum* groups well implicates the antioxidant status in the *C. verum*. In previous studies, the increase in reactive oxygen species level was associated with the infiltration rate of neutrophils in the high-glucose environment of diabetic wounds (Bitar and Al-Mulla 2012; Maggio et al. 2013). Antioxidant property of cinnamon is a key factor for the improvement of wound healing (Kamath et al. 2003; Farahpour and Habibi 2012). Faulted antioxidant defence system under diabetic condition has been accepted, and plant derivatives (extracts, essential oils and active compounds) have been suggested for improving the antioxidant system (Eo et al. 2016). The oxidative stress in the wound area increases the damage of proteins, nucleotides and lipid levels (Sharma et al. 2012; Modarresi et al. 2019). The significant changes in the antioxidant profile are associated with the increase in the levels of MDA content, which is mainly attributed to the lipid peroxidation ratio (Sharma et al. 2012). It can be concluded that *C. verum* prevents the cellular DNA, RNA and protein damage by up-regulating the antioxidant status involved in the healing process (Figure 5). On the other hand, antioxidants are involved in the wound healing process by reducing inflammation (Süntar et al. 2012; Farahpour et al. 2015). In the following, the inflammatory activity of *C. verum* is elucidated.

The inflammation phase is a major step in eliminating the cellular debris from tissue as well as extensive response to microbial infection (Landén et al. 2016; Manzuoerh et al. 2019). Neutrophils, macrophages and lymphocytes infiltrate the injured area during the inflammatory stage (Qing 2017; Modarresi et al. 2019). Our microscopic analyses confirmed the infiltration of the inflammatory cells into the wound site in the *C. verum*-treated group on day 7 after wound creation. The cinnamon water extract inhibits monocyte-to-macrophage differentiation (Bao et al. 2009). Anti-inflammatory effect of *C. verum* can be attributed to its compounds including cinnamaldehyde (Yang et al. 2016) 2-hydroxycinnamaldehyde (Lee et al. 2005) and quercetin (Caddeo et al. 2014). It can be stated that *C. verum* contains a high number of anti-inflammatory agents, resulting in the shortening of the inflammatory stage and starting the proliferation stage.

Rapid cellular proliferation and differentiation are necessary in shortening the healing time (Oryan et al. 2015; Karimzadeh and Farahpour 2017). Controlled apoptosis and up-regulated revascularization are associated with the increase in collagen deposition which accelerates the healing process (Wu and Chen 2014; Manzuoerh et al. 2019). The increase in collagen biosynthesis in the *C. verum*-treated accelerates proliferation. Also accepted is the role of fibroblasts and fibrocytes in the synthesis of collagen (Li et al. 2006; Stunova and Vistejnova 2018). It seems the increased collagen biosynthesis is related to cyclin D1 expression in the *C. verum*-treated animals, controlling the progression through the G_1_ phase of the cell cycle in fibroblasts and mammary epithelial cells. The cell cycle transition via G1 and its over-expression accelerates the cell cycle via progressing the G1 phase of the cell cycle during cell proliferation, and further stimulates contact-independent cellularity (Tashiro et al. 2007). On the basis of our findings, topical administration of *C. verum* increased fibroblasts and fibrocytes distribution by cyclin D1. The RT-PCR analyses showed that the mRNA level of cyclin D1 increased in the *C. verum*-treated animals. Thus, it can be logically accepted that the administration of *C. verum* promotes the proliferation stage by up-regulating the cellular proliferation via increased cyclin D1 expression. In addition, cyclin D1 accelerates the cellular migration in injured sites and subsequently initiates angiogenesis (Roovers et al. 2003). Cyclin D1 promotes cell migration as well as angiogenesis through inhibiting the expression of thrombospondin-1 as the main agent involved in inhibiting the angiogenesis (Wang et al. 2004). Our histomorphometric findings confirmed that the administration of *C. verum* increased vascularization compared to the diabetic non-treated group, suggesting the role of *C. verum* in up-regulating the cyclin D1 and subsequent angiogenesis.

The other factor involved in proliferative cell models is GLUT-1 whose expression was increased in the *C. verum* groups. RT-PCR results also showed that after 7 d, the administration of *C. verum* significantly increased the mRNA level of GLUT-1. Up-regulation of GLUT-1 can be the reason behind the improvement in wound healing in diabetic rats (Bonab and Farahpour 2017; Yu et al. 2017; Daemi et al. 2019). Velander et al. (2008) observed that delayed wound healing in the experimentally-induced diabetes was not induced by local high-glucose concentration alone. Cao et al. (2010) showed that cinnamon extract administration resulted in glucose transporter regulation and insulin-signalling gene expression. Moreover, it has been shown that cinnamaldehyde, as the main cinnamon extract medical agent in the cinnamon extract, up-regulates the GLUT-4 expression in the mouse muscle cells (Nikzamir et al. 2014); therefore, it could be concluded that *C. verum* may facilitate the glucose transport pathway via cell membrane by up-regulating the GLUT-1 expression.

Our RT-PCR analyses further showed that *C. verum* significantly increased IGF-1 expression. Indeed, the expression of IGF-1 in wounds mainly depends on the type of the induced wound (Amiri et al. 2014). Administration of IGF-1 has improved insulin sensitivity and lowered the risk of type 2 diabetes (Chiasson and Rabasa-Lhoret 2004). A significant reduction has been also reported in IGF-1 of the diabetic wounds’ fluid (Velander et al. 2008). Furthermore, locally delivered IGF-1 accelerates healing in senescent mice (Lisa et al. 2011) and the proliferation and migration of keratinocyte and fibroblast cells in *in vitro* assessment (Lisa et al. 2011; O’Neill et al. 2015). IGF-1 also promotes glucose transport in short-term periods during the wound healing process (Tsuboi et al. 1995; Daemi et al. 2019). Therefore, *C. verum* increased the proliferation of fibroblasts and glucose uptake in the keratinocytes, and finally accelerated wound healing during diabetes through up-regulating the IGF-1 expression.

## Conclusions

Administration of an ointment prepared from cinnamon extract promoted cellular proliferation by increased cyclin D1 expression, especially in fibroblasts, fibrocytes and keratinocyte cells. It also increased the GLUT-1 and IGF-1 expression, and improved cellular energy uptake and proliferative properties. Therefore, it is recommended that *C. verum* hydroethanolic extract be applied in commercial ointments to improve wound healing.

## References

[CIT0001] Aghdam SY, Eming SA, Willenborg S, Neuhaus B, Niessen CM, Partridge L, Krieg T, Bruning JC. 2012. Vascular endothelial insulin/IGF-1 signaling controls skin wound vascularization. Biophys Res Commun. 421:197–202. 10.1016/j.bbrc.2012.03.134 22503682

[CIT0002] Amiri FT, Fathabadi FF, Rad MM. 2014. The effects of insulin-like growth factor-1 gene therapy and cell transplantation on rat acute wound model. Iran Red Crescent Med J. 16:1–7. 10.5812/ircmj.16323 PMC4270678 25558384

[CIT0003] Bao P, Kodra A, Tomic-Canic M, Golinko MS, Ehrlich HP, Brem H. 2009. The role of vascular endothelial growth factor in wound healing. J Surg Res. 153:347–358. 19027922 10.1016/j.jss.2008.04.023 PMC2728016

[CIT0004] Bitar MS, Al-Mulla F. 2012. ROS constitute a convergence nexus in the development of IGF1 resistance and impaired wound healing in a rat model of type 2 diabetes. Dis Model Mech. 5:375–388. 22362362 10.1242/dmm.007872 PMC3339831

[CIT0005] Blakytny R, Jude E. 2006. The molecular biology of chronic wounds and delayed healing in diabetes. Diabet Med. 23:594–608. 16759300 10.1111/j.1464-5491.2006.01773.x

[CIT0006] Bonab FS, Farahpour MR. 2017. Topical co-administration of *Pistacia atlantica* hull and *Quercus infectoria* gall hydroethanolic extract improves wound-healing process. Comp Clin Pathol. 26:885–892.

[CIT0007] Caddeo C, Díez-Sales O, Pons R. 2014. Topical anti-inflammatory potential of quercetin in lipid-based nanosystems: *in vivo* and *in vitro* evaluation. Pharm Res. 1:959–968. 10.1007/s11095-013-1215-0 24297068

[CIT0008] Cao H, Graves DJ, Anderson RA. 2010. *Cinnamon* extract regulates glucose transporter and insulin-signaling gene expression in mouse adipocytes. Phytomedicine. 17:1027–1032. 20554184 10.1016/j.phymed.2010.03.023

[CIT0009] Chiasson JL, Rabasa-Lhoret R. 2004. Prevention of type 2 diabetes: insulin resistance and beta-cell function. Diabetes. 1:38–44. 10.2337/diabetes.53.suppl_3.s34 15561919

[CIT0010] Daemi A, Farahpour MR, Oryan A, Karimzadeh S, Tajer E. 2019. Topical administration of hydroethanolic extract of *Lawsonia inermis* (henna) accelerates excisional wound healing process by reducing tissue inflammation and amplifying glucose uptake. Kaohsiung J Med Sci. 35:24–32. 30844141 10.1002/kjm2.12005 PMC11900754

[CIT0011] Eo H, Lee H-J, Lim Y. 2016. Ameliorative effect of dietary genistein on diabetes induced hyper-inflammation and oxidative stress during early stage of wound healing in alloxan induced diabetic mice. Biochem Biophys Res Commun. 478:1021–1027. 27431618 10.1016/j.bbrc.2016.07.039

[CIT0012] Farahpour MR, Habibi M. 2012. Evaluation of the wound healing activity of an ethanolic extract of Ceylon cinnamon in mice. Veterinarni Medicina. 57:53–57.

[CIT1001] Farahpour MR, Hesaraki S, Faraji D, Zeinalpour R, Aghaei M. 2017. Hydroethanolic Allium sativum extract accelerates excision wound healing: evidence for roles of mast-cell infiltration and intracytoplasmic carbohydrate ratio. Braz J Pharm Sci. 53(1):e15079.

[CIT2001] Farahpour MR, Mirzakhani N, Doostmohammadi J, Ebrahimzadeh M. 2015. Hydroethanolic Pistacia atlantica hulls extract improved wound healing process; evidence for mast cells infiltration, angiogenesis and RNA stability. Inter J Surg. 17:88–98. 10.1016/j.ijsu.2015.03.019 25849027

[CIT0013] Farahpour MR, Vahid M, Oryan A. 2018. Effectiveness of topical application of ostrich oil on the healing of *Staphylococcus aureus* and *Pseudomonas aeruginosa*-infected wounds. Connect Tissue Res. 59:212–222. 28682114 10.1080/03008207.2017.1350174

[CIT0014] Kamath JV, Rana AC, Chowdhury AR. 2003. Pro-healing effect of *Cinnamomum zeylanicum* bark. Phytother Res. 17:970–972. 13680838 10.1002/ptr.1293

[CIT0015] Karimzadeh S, Farahpour MR. 2017. Topical application of *Salvia officinalis* hydroethanolic leaf extract improves wound healing process. Indian J Exp Biol. 55:98–106. 30183236

[CIT0016] Landén NX, Li D, Ståhle M. 2016. Transition from inflammation to proliferation: a critical step during wound healing. Cell Mol Life Sci. 73:3861–3885. 27180275 10.1007/s00018-016-2268-0 PMC5021733

[CIT0017] Lee SH, Lee SY, Son DJ, Lee H, Yoo HS, Song S, Oh KW, Han DC, Kwon BM, Hong JT. 2005. Inhibitory effect of 2′-hydroxycinnamaldehyde on nitric oxide production through inhibition of NF-κB activation in RAW 264.7 cells. Biochem Pharmacol. 69:791–799. 15710356 10.1016/j.bcp.2004.11.013

[CIT0018] Li Z, Wang C, Jiao X, Lu Y, Fu M, Quong AA, Dye C, Yang J, Dai M, Ju X, et al. 2006. Cyclin D1 regulates cellular migration through the inhibition of thrombospondin 1 and ROCK signaling. Mol Cell Biol. 26:4240–4256. 16705174 10.1128/MCB.02124-05 PMC1489104

[CIT0019] Lisa M, Haleagrahara N, Chakravarthi S. 2011. Insulin-like growth factor-1 (IGF-1) reduces is changes and increases circulating angiogenic factors in experimentally-induced myocardial infarction in rats. Vasc Cell. 3:13–20. 21651821 10.1186/2045-824X-3-13 PMC3131242

[CIT0020] Maggio M, De Vita F, Lauretani F, Buttò V, Bondi G, Cattabiani C, Nouvenne A, Meschi T, Dall'Aglio E, Ceda GP. 2013. IGF-1, the cross road of the nutritional, inflammatory and hormonal pathways to frailty. Nutrients. 5:4184–4205. 24152751 10.3390/nu5104184 PMC3820068

[CIT0021] Mang B, Wolters M, Schmitt B, Kelb K, Lichtinghagen R, Stichtenoth DO, Hahn A. 2006. Effects of a cinnamon extract on plasma glucose, HbA1c, and serum lipids in diabetes mellitus type 2. Eur J Clin Invest. 36:340–344. 16634838 10.1111/j.1365-2362.2006.01629.x

[CIT0022] Manzuoerh R, Farahpour MR, Oryan A. 2019. Effectiveness of topical administration of *Anethum graveolens* essential oil on MRSA-infected wounds. Biomed Pharmacother. 1:1650–1658. 10.1016/j.biopha.2018.10.117 30551419

[CIT0023] Modarresi M, Farahpour MR, Baradaran B. 2019. Topical application of *Mentha piperita* essential oil accelerates wound healing in infected mice model. Inflammopharmacology. 27:531–537. 29980963 10.1007/s10787-018-0510-0

[CIT0024] Mohammadi A, Mohammad-Alizadeh-Charandabi S, Mirghafourvand M, Javadzadeh Y, Fardiazar Z, Effati-Daryani F. 2014. Effects of cinnamon on perineal pain and healing of episiotomy: a randomized placebo-controlled trial. J Integr Med. 12:359–366. 25074885 10.1016/S2095-4964(14)60025-X

[CIT0025] Nikzamir A, Palangi A, Kheirollaha A, Tabar H, Malakaskar A, Shahbazian H, Fathi M. 2014. Expression of glucose transporter 4 (GLUT4) is increased by cinnamaldehyde in C2C12 mouse muscle cells. Iran Red Crescent Med J. 16:e13426. 24719730 10.5812/ircmj.13426 PMC3965863

[CIT0026] O’Neill BT, Lauritzen HP, Hirshman MF. 2015. Differential role of insulin/IGF-1 receptor signaling in muscle growth and glucose homeostasis. Cell Rep. 11:1220–1235. 25981038 10.1016/j.celrep.2015.04.037 PMC4449334

[CIT0027] Oryan A, Alemzadeh E. 2017. Effects of insulin on wound healing: a review of animal and human evidences. Life Sci. 174:59–67. 28263805 10.1016/j.lfs.2017.02.015

[CIT0028] Oryan A, Mohammadalipour A, Moshiri A, Tabandeh MR. 2015. Avocado/soybean unsaponifiables: a novel regulator of cutaneous wound healing, modelling and remodelling. Int Wound J. 12:674–685. 24321012 10.1111/iwj.12196 PMC7950965

[CIT0029] Qing C. 2017. The molecular biology in wound healing & non-healing wound. Chinese J Traumatol. 20:189–193. 10.1016/j.cjtee.2017.06.001 PMC5555286 28712679

[CIT0030] Roovers K, Klein EA, Castagnino P, Assoian RK. 2003. Nuclear translocation of LIM kinase mediates Rho-Rho kinase regulation of cyclin D1 expression. Dev Cell. 5:273–284. 12919678 10.1016/s1534-5807(03)00206-5

[CIT0031] Sharma P, Jha AB, Dubey RS. 2012. Reactive oxygen species, oxidative damage, and antioxidative defense mechanism in plants under stressful conditions. J Bot. 24:1–26.

[CIT0032] Stunova A, Vistejnova L. 2018. Dermal fibroblasts – a heterogeneous population with regulatory function in wound healing. Cytokine Growth Factor Rev. 39:137–150. 29395658 10.1016/j.cytogfr.2018.01.003

[CIT0033] Süntar I, Akkol EK, Nahar L, Sarker SD. 2012. Wound healing and antioxidant properties: do they coexist in plants? Free Radic Antioxidant. 2:1–7.

[CIT0034] Tashiro E, Tsuchiya A, Imoto M. 2007. Functions of cyclin D1 as an oncogene and regulation of cyclin D1 expression. Cancer Sci. 98:629–635. 17359287 10.1111/j.1349-7006.2007.00449.x PMC11159462

[CIT0035] Tsuboi R, Shi C-M, Sato C, Cox GN, Ogawa H. 1995. Co-administration of insulin-like growth factor (IGF)-I and IGF-binding protein-1 stimulates wound healing in animal models. J Invest Dermatol. 104:199–203. 7530269 10.1111/1523-1747.ep12612755

[CIT0036] Velander P, Theopold C, Hirsch T, Bleiziffer O, Zuhaili B, Fossum M, Hoeller D, Gheerardyn R, Chen M, Visovatti S, et al. 2008. Impaired wound healing in an acute diabetic pig model and the effects of local hyperglycemia. Wound Repair Regen. 16:288–293. 18318812 10.1111/j.1524-475X.2008.00367.x

[CIT0037] Wang C, Li Z, Fu M, Bouras T, Pestell RG. 2004. Signal transduction mediated by cyclin D1: from mitogens to cell proliferation: a molecular target with therapeutic potential. Cancer Treat Res. 119:217–237. 15164880 10.1007/1-4020-7847-1_11

[CIT0038] Wu YS, Chen SN. 2014. Apoptotic cell: linkage of inflammation and wound healing. Front Pharmacol. 5:1–6. 24478702 10.3389/fphar.2014.00001 PMC3896898

[CIT0039] Yang D, Liang X-C, Shi Y, Sun Q, Liu D, Liu W, Zhang H. 2016. Anti-oxidative and anti-inflammatory effects of cinnamaldehyde on protecting high glucose-induced damage in cultured dorsal root ganglion neurons of rats. Chin J Integr Med. 22:19–27. 26577110 10.1007/s11655-015-2103-8

[CIT0040] Yu T, Gao M, Yang P, Pei Q, Liu D, Wang D, Zhang X, Liu Y. 2017. Topical insulin accelerates cutaneous wound healing in insulin-resistant diabetic rats. Am J Transl Res. 9:4682–4693. 29118927 PMC5666074

